# The Pathology of Appendicitis

**Published:** 1896-05-30

**Authors:** H. S. Wansbrough Jones


### THE PATHOLOGY OF APPENDICITIS.

By H. S. Wansbrough Jones, M.B. Edin., B.Sc., Lond.

(*Continued from page* 124.)

It will be well, before describing the general course of the inflammatory processes, to say something of causes which may predispose to this condition. Now, it will be evident at once that these may be classified as (1) traumatism; (2) gastro-intestinal disturbance; (3) constitutional conditions. Any traumatism which can cause abrasion of surface, and so allow of the entrance of organisms, particularly if the lymphoid tissue be damaged, or the arterial supply interfered with, would obviously lead to an attack of appendicitis. Among such we may have injuries from twists and blows, strangulations, or abrasions and occlusions from appendicular calculi. Morris asserts that twists of the appendix and its mesentery may sometimes cause serious interference with the arterial and venous circulation. The appendix is not infrequently found both in inguinal and femoral herniæ, and in these positions may become injured and the seat of inflammation. The possibility of internal strangulation in the retro-cæcal form has been mentioned above; there are many examples on record of the existence of this condition. But perhaps traumatism is most commonly produced by appendicular calculi. Supposing a calculus has formed in the appendix, it is clear that the danger from blows or twists will be immensely increased, and also that violent peristaltic efforts made to expel it may cause wounding of the walls; but there is also danger from the possibility of occlusion, in which case the secretions collecting behind the body would make the whole structure tense, thus causing injury to the lymphoid tissue, and possibly injury and occlusion of the solitary terminal artery. This is certainly one of the ways in which gangrene of the appendix is produced.

The exact influence of gastro-intestimal disturbances is doubtful, but there can be no question that it is considerable, and it is probably owing to disturbances of this nature interfering with the regular expulsion of appendicular contents that the formation of calculi is due. But whether this is so or not, when once a calculus is formed an effort will be made to expel it. This may take place easily by the pressure of retained secretion behind it, but it may be that the relation of the size of the calculus to the calibre of the appendix is such that only a marked increase of peristaltic effort can effect this expulsion. The violent peristalsis produced by irritants in the small intestine will extend to the appendix. So that it is easy to see that given the presence in the appendix of a calculus not easily expelled, gastro-intestinal disturbances will lead to violent efforts to produce this in the appendix, and so an attack of appendicular colic will result. The relation of appendicitis to general conditions, unless it be tuberculosis, is even more doubtful. Probably the most interesting is the connection with the so-called uric acid diathesis.

In a paper published in the *British Medical Journal* of April 23rd, 1892, Sutherland gives some interesting examples of this relation. Morris, whose very interesting book on appendicitis appeared last year, mentions the fact that appendicitis has been not infrequently diagnosed as "gravel," and it is not uncommon to find symptoms of appendicitis associated with those of chronic rheumatism. These facts are particularly interesting in view of the association which Horbacszewski and others have lately established between uric acid conditions and the leucocytes and lymphoid tissues.

It only remains to consider the actual local processes at work. Appendicitis is an infective, exudative inflammation of the appendix, due to any of those causes enumerated above which can injure its tissues, or cause an erosion of the mucosa, and so allow the entrance of micro-organisms. When once these have entered the lymphoid tissue, the struggle between them and the leucocytes begins. As a part of the inflammatory process there is exudation into the lymphoid tissue and connective tissue which rapidly swells: owing to the inelastic nature of the outer tube, the inner tube can only expand inward, and so exclude the lumen of the appendix. Obstruction of the artery or its small branches soon follows. This may in some cases be due to the twisting of the mesappendix, or direct pressure from the exudate, but is also often due to obliterative endarteritis. As there is no collateral blood supply, this condition, if it affects the main artery, may cause complete gangrene of the appendix, and often, by its effect on the smaller branches, causes small local patches of gangrene. In any case, it is one of the most important factors in the production of serious effects, for it prevents the supply of fresh leucocytes to the infected site, and so allows the organisms to get mastery in the struggle going on there.

If the injury and inflammation be not severe, there will be simply more or less destruction of the mucous and lymphoid coats; ulceration, which may heal quickly and cause no serious trouble, though it may lead to subsequent stricture. There may, on the other hand, be all degrees of severity, leading to destruction of all the coats of the appendix, and so causing perforation, or the infiltration and exudation, and the action of the toxines may be so intense as to cause rapid and complete gangrene of the organ. Extension of the veins may lead to infective thrombosis, and so may cause pylephlebitis and secondary abscesses in the liver or elsewhere; or the infection may spread in the lymphatic and connective tissue planes, and lead to abscesses in the pelvis, thigh, and buttock, or pass upwards to the region of the kidney.

If perforation occur, the peritoneum immediately becomes infected, a complication fraught with danger, but fortunately provided against by its abundant vascularity, which brings multitudes of leucocytes to deal with the organisms and their toxines. In the majority of cases adhesions soon form, which wall off the infected appendix, and whatever may have escaped from it, from the main peritoneal cavity. In some cases, however, the infection is not so limited, and a general purulent peritonitis results.

It will be noted that the chief facts which have been added to the pathology of this disease are the importance of the lymphoid coat of the appendix, the method of formation of the appendicular calculi, the knowledge of the causes and mechanism of internal strangulation and other injuries, and the significance and influence of its arterial supply by a solitary terminal artery and the obliterative arteritis produced in the course of the disease.

Works Consulted —Berry's "Pathology of the Vermiform Appendix," Journal of Pathology and Bacteriology, April, 1895. "Anatomy of the Vermiform Appendix," Anatomischer Anzeiger, X., November 24, 1893. "Anatomy of the Cæcum," Anatomischer Anzeiger, X. 13, 1895. Morris, "Lectures on Appendicitis," 1895. Talamon, "Appendicitis and Perityphilitis." Kelymack, "The Pathology of the Vermiform Appendix," 1893. "Sutherland on Some Systems Associated with the Uric Acid Diathesis in Children," B,M.J., April 23, 1892.

